# Association of *P73* polymorphisms with susceptibilities of cervical carcinoma: a meta-analysis

**DOI:** 10.18632/oncotarget.18164

**Published:** 2017-05-24

**Authors:** Xianghua Liang, Bingxiang Chen, Jianxin Zhong

**Affiliations:** ^1^ Department of Gynecology and Obstetrics, Qidong People's Hospital, Jiangsu, China; ^2^ Department of Gynecology and Obstetrics, Affiliated Hospital of Nantong University, Jiangsu, China

**Keywords:** P73 gene, cervical cancer, meta analysis

## Abstract

**Objective:**

The relation between *P73* gene polymorphism and cervical cancer has not been determined. At present, we utilized a meta-analysis method to elucidate the association between *P73* and cervical cancer.

**Results:**

The present study included 635 patients with cervical cancer and 998 cancer-free control subjects. Using meta-analysis, we found a significant association of P73 genetic polymorphism with cervical cancer in a recessive model [OR = 0.91, 95% CI: 0.84−0.98; *P* = 0.02.]. However, this association was not find in a dominant model [OR = 0.76, 95% CI (0.45−1.27); *P* = 0.29], in a co-dominant model [OR = 1.01; 95% CI: 0.98–1.04, *P* = 0.56] or in an allelic model [OR = 0.97, 95% CI: 0.93−1.00; *P* = 0.09].

**Materials and Methods:**

To further evaluate the relation between the *P73* gene polymorphism and cervical cancer, we selected 5 case-control studies related to *P73* gene polymorphism and cervical cancer by searching CNKI, VIP, WanFang, PubMed and EMbase database. We utilized *Q*-test and I^2^ test to test the heterogeneity between each study. The fixed effects model was utilized to calculate the odds ratio (OR) and its 95% confidence interval.

**Conclusions:**

Our results suggest that *P73* gene polymorphism was associated with the risk of cervical cancer. However, our conclusion still requires large sample size of case-control studies or cohort studies to further confirm this result.

## INTRODUCTION

Cervical cancer is a common gynecological malignancy with high mortality [1], which is a serious threat to women’s health and life [2]. The current evidences suggest that the occurrence and development of cervical cancer is associated with activation of oncogenes [3–4]. Recently, *P73* gene is identified a candidate gene of tumor suppressor and plays an important role in the development of many tumors [5–6]. A number of studies have shown that *P73* gene polymorphisms are associated with the risk for cervical cancer, but the results are not conclusive. Jha et al. found *P73* polymorphism was associated with the risk of cervical cancer in a Japanese population [7]. However, the subsequent studies performed in Caucasian [8] and in China [9] did not shown association of P73 polymorphism with cervical cancer. This inconsistency may result from the small sample size in each study. Therefore, we collected almost all published case-control studies to perform a meta-analysis to clarify the relation between *P73* gene polymorphism and cervical cancer.

## RESULTS

### Study identification

**Figure 1:**
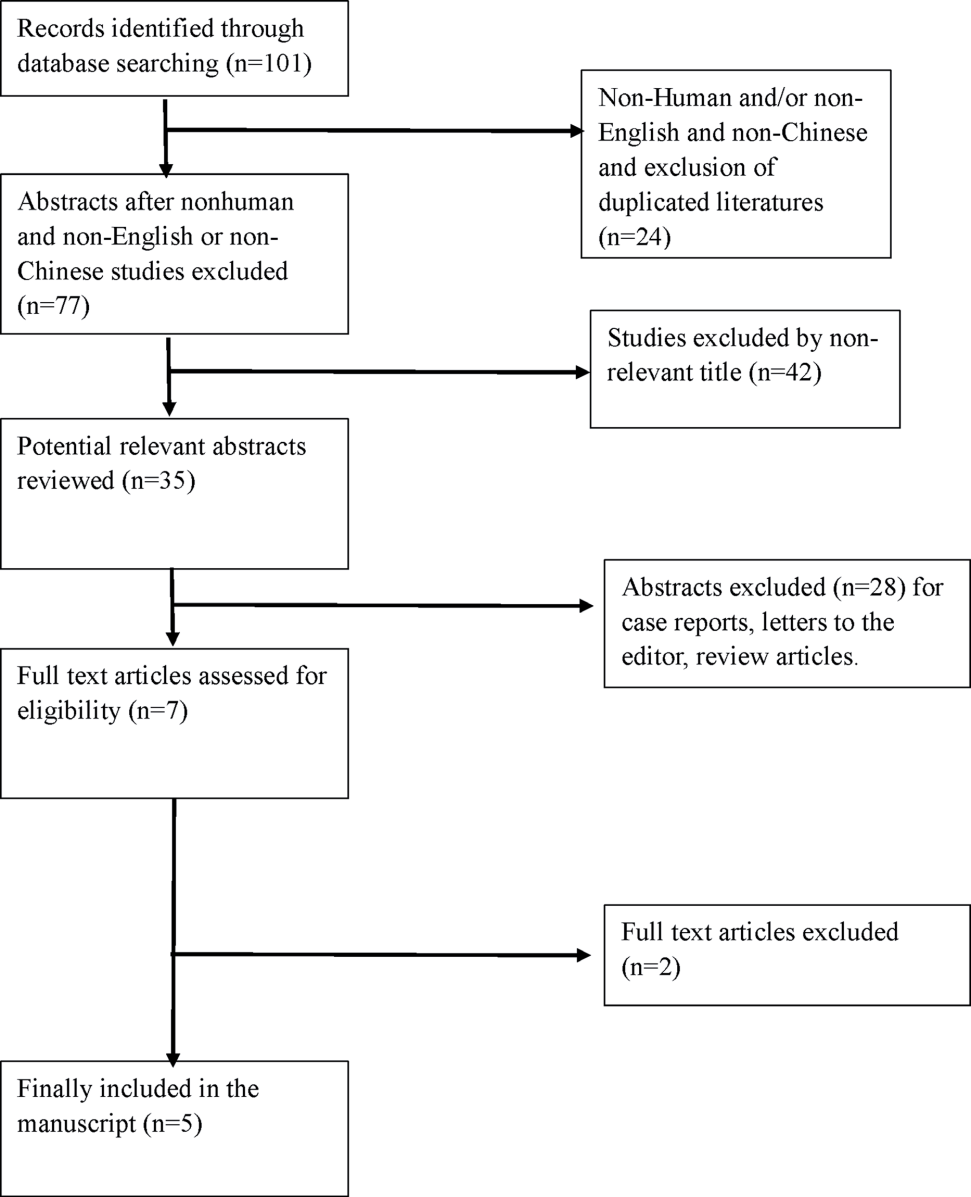
Flow diagram of the literature search and study selection

As shown in Figure 1, 101 literatures including 88 English literatures and 13 Chinese literatures were reviewed preliminarily. 89 literatures were excluded because of duplicate publication and nonclinical-based data. 7 studies were further excluded because of no control subjects. Therefore, a total of 5 literatures [7–11] were included in the final analysis, with a total of 635 patients with cervical cancer and 998 cancer-free control subjects. The characteristics of included studies were shown in Table 1.

**Table 1:** Characteristics of included studies

Authors	Publication year	Country	Ethnicity	Genotype (case/control )			HWE
				GC/GC	GC/AT	AT/AT	Yes
Craveiro et al.	2012	Portugal	Caucasus	95/119	38/48	8/9	Yes
Jha et al.	2012	India	Asian	71/77	28/19	2/4	Yes
Niwa et al.	2004	Japan	Asian	57/270	52/150	3/22	Yes
Zheng et al.	2008	China	Asian	71/77	28/19	2/4	Yes
Feng et al.	2017	China	Asian	103/114	67/55	10/11	Yes

### Quantitative synthesis

**Figure 2:**
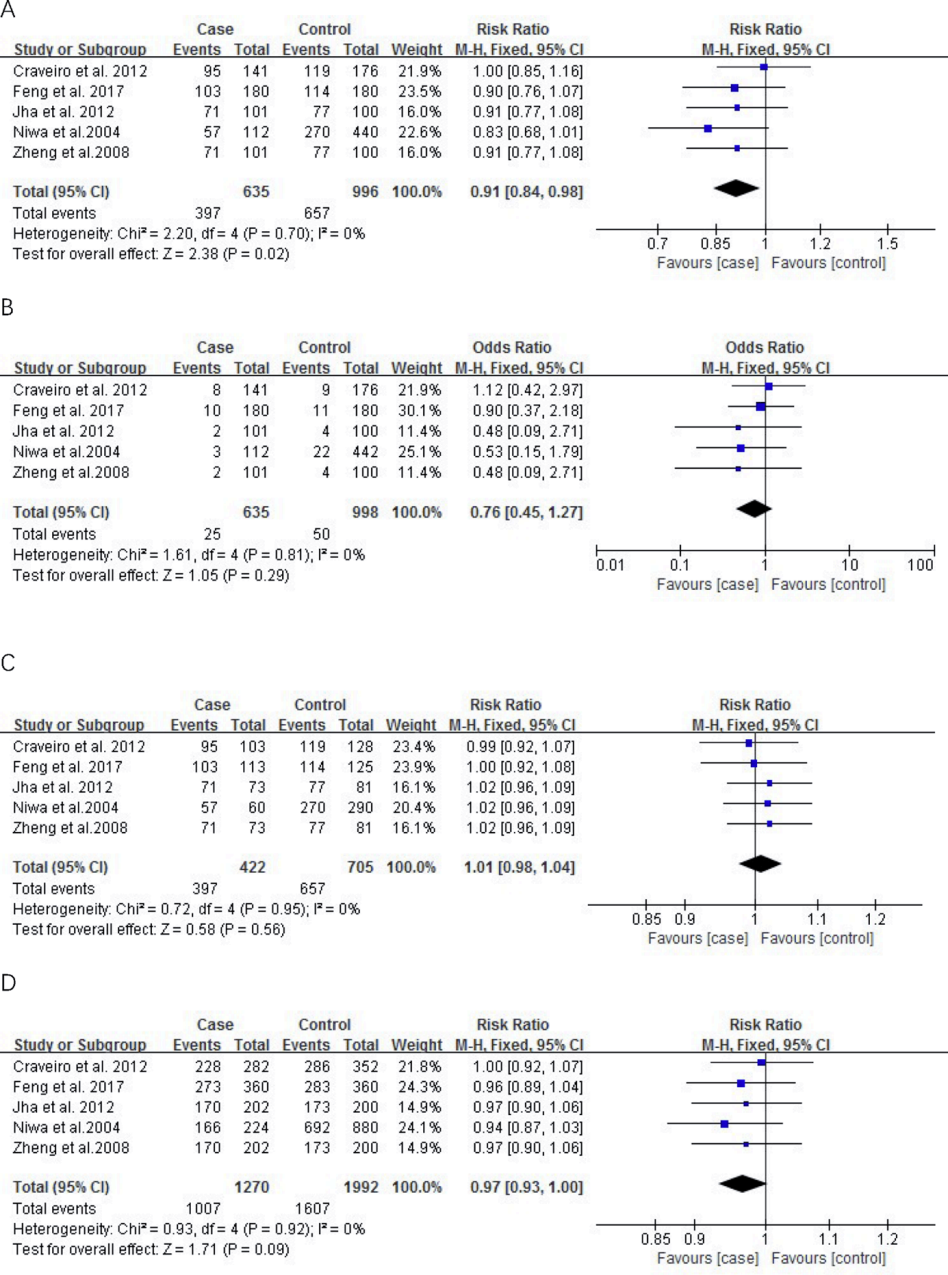
Meta-analysis of genetic polymorphism and cervical cancer (**A**) Recessive model; (**B**) Dominant model; (**C**) Co-dominat model; (**D**) Allelic model.

The result of meta-analysis of studies on the correlation between cervical cancer and *P73* gene polymorphism in 5 case-control studies as shown in Figure 2 which include the number of case and control groups, weight, OR value, and 95% CI. The heterogeneity test of the various studies did not shown heterogeneous results. Therefore, we used the fixed effects model in the analysis. Overall, the association of *P73* with risk of cervical cancer was observed in a recessive model [OR = 0.91, 95% CI: 0.84–0.98, *P* = 0.02.]. However, this association was not find in a dominant model [OR = 0.76, 95% CI (0.45–1.27); *P* = 0.29], in a co-dominant model [OR = 1.01; 95%CI: 0.98–1.04, *P* = 0.56] or in an allelic model [OR = 0.97, 95% CI: 0.93–1.00; *P* = 0.09].

### Publication bias analysis

**Figure 3:**
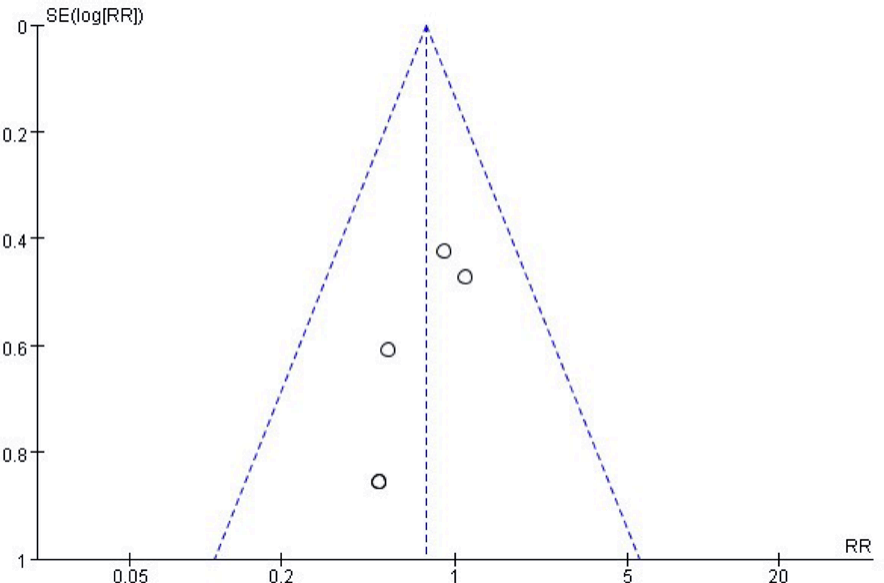
Publication bias analysis

We utilized the RevMan 5.0 software to analyze the publication bias. The funnel plot ( Figure 3) shows that the points are evenly distributed and symmetrical, and most of the points are within the 95% confidence interval, the shape of funnel plot shows no obvious asymmetry. It indicates that there is no publication bias in the present study, and the result is credible.

## DISCUSSION

In the present study, we performed a meta-analysis to evaluate the association of *P73* polymorphism with cervical cancer. We find a significant association of *p73* gene polymorphism with cervical cancer in a recessive model but not in a dominant, a co-dominant, or an allelic model. This study clarified the association between *P73* polymorphism and cervical cancer.

The *P73* gene is located at the 1p36 position on the human chromosome, and the P73 protein activates transcription of a number of P53-responsive genes to participate in cell cycle regulation, DNA repair and apoptosis, and in the same way as P53 by inducing apoptosis or G1 phase of cell arrest to inhibit cell proliferation [12–16]. Previously, several studies related to the relation between *P73* and cervical cancer risk has been reported, however, the results were inclusive. In a Japanese population, GC /AT genotype and AT/AT genotype were associated with the occurrence of cervical cancer [7]. A study of white women in Portugal reported that GC / AT genotype was associated with low age at menarche [8]. However, a recent study of Caucasian showed that *P73* gene GC / AT polymorphism was associated with cervical intraepithelial neoplasia (HSIL) and was not associated with cervical cancer [9]. Also, a recent study including Uygur population indicated that *P73* gene GC/AT polymorphism was not associated with cervical cancer [11]. This inconsistency may result from the small sample size and the different experimental methods. The present study has shown that *p73* gene polymorphism was associated with the susceptibility of cervical cancer. However, there is still a need for further research and screening of etiological relations between the functional polymorphism loci of the p73 gene and the susceptibility of cervical cancer.

Several limitations should be considered when interpreting these results. Firstly, all literatures involved in our study are of different languages. Secondly, the different research methods may increase the heterogeneity of these studies. Finally, a small sample of some included case-control studies may reduce the test power. To some extent, it reduced the reliability and comprehensiveness of the results of this systemic evaluation.

In conclusion, the present study suggested that there is an association between P73 gene polymorphism and cervical cancer.

## MATERIALS AND METHODS

### Literatures collection and screening

To identify all the articles that explored the association of *P73* polymorphisms with cervical cancer risk, we conducted a computerized literature search of MEDLINE, EMBase, Chinese Biomedical Literature Database (VIP), Chinese CNKI, and Wanfang database using the terms “cervical cancer (Mesh),” “P73”, “gene polymorphism,” or “SNP” without any restriction on language or publication year. By means of online retrieval and literature review, references obtained using the above-mentioned databases were reviewed again to ensure that no relevant studies are missed.

### The inclusion and exclusion criteria of literatures

All the included studies must meet to the following criteria: (1) independently published case-control or cohort studies on the relation between *P73* polymorphism and cervical cancer; (2) with comprehensive statistical indicators directly or indirectly: OR or RR (relative risk) values and 95% CI (confidence interval); and (3) similar themes and methods, that is, case-control or cohort studies about the relation of the *P73* gene polymorphism and cervical cancer. The literatures were excluded if relevant data are not available or there is heterogeneity of gene polymorphism in the control population.

### Quality assessment and data extraction

Two reviewers independently evaluated the research design, enrolled patients, observation results of the literature, and selected trials according to the above-mentioned inclusion criteria. Inconsistencies were resolved through discussion. We used the Cochrane Handbook 5.2 quality evaluation criteria to assess the methodological quality of included studies. To determine the quality of data by the quality of ultimately determined literature, the useless ones will be excluded, such as studies that have been reported repeatedly and those with poor quality or less information and have special selection of laboratory sample; relevant data were extracted from included literatures.

### Statistical analysis

We performed the present meta-analysis utilized RevMan 5.2 software which provided by the Cochrane Collaboration. *Q*-test and *I*^2^ test was used to examine the heterogeneity between each study. We used odds ratio (OR) for efficacy analysis statistics. In the present study, we selected the fixed effects model to merge the OR. Analysis of sensitivity includes the difference of point estimation and confidence intervals of the combined effects value of different models to observe whether it changes the result. To test the publication bias, we utilized the RevMan 5.2 statistical software to make the funnel plot. *P* < 0.05 was considered as a significant difference.
